# On the Efficacy and Fallacy of New Remedies

**Published:** 1820-07

**Authors:** 


FOR THE LONDON MEDICAL AND PHYSICAL JOURNAL.

### *On the Efficacy and Fallacy of New Remedies.* By Dr. Kinglake.

The materia medica has already been carried to a great extent. The proposal of new remedies has in all ages been received with indulgence, and in numerous instances an intemperate earnestness has been shown for their adoption. Although the medicinal substances that have been progressively admitted, and catalogued as possessing sufficient remedial powers to entitle them to such an adoption, have been vastly extensive and various, yet there is still manifested an anxious desire for augmenting the number. This arises from the strong recommendation with which new remedies are usually introduced, not abiding the test of experience. They are very generally found, on trial, to be at least wanting in the efficacy ascribed to them, if not altogether unworthy of any degree of reasonable dependence. This has proved a source of serious injury and disappointment in the practice of medicine. The instruments of medicine are of as much importance to the medical practitioner as suitable tools are to the expert mechanic: in neither case can the desired object be effected without their respective aid. The indications for the use of medicine are numerous, and are required to be fulfilled by appropriate means. Much may always be accomplished by dietetic and other domestic regulations with almost infallible certainty. Positive and negative directions in medical practice for obtaining given results, are amongst the most valuable resources of the healing art. It often happens, however, that, with the best-poised consideration of all the relative circumstances that may be adduced for and against a medical view of a disease, and all the benefits accruing from this knowledge, that the relief which may be urgently required cannot be procured by any known medicinal influence. The aid that may be wanted is always sufficiently evident, but how it is to be afforded is the difficulty. *Hoc opus hic labor est.* In this solicitude for applying an adequate remedy, every instance that presents of furnishing superior medicinal powers is eagerly embraced, and with hopes and expectations that oftener disappoint than realize the promised advantage.

It is less to be wondered that new medicines should fail in verifying the high character at first given of their efficacy, than that, under the usual precipitate and unguarded commendation of their powers, they should be ultimately admitted into the lists of the materia medica.

It is quite notorious, that many of the officinal articles adopted and sanctioned by late revisions of the most respectable Pharmacopœias, are destitute of any certain powers that could be beneficially employed under any known circumstances of disease. The instances in which many of them have been at all availing are so vague and indefinite, that better authority would seem to be necessary to warrant their unqualified adoption. It is easier to object to the inefficacy or fallacy of medicinal substances, than to substitute what would possess more precise and efficient virtue; yet that should not be regarded as resting-ground which really has no sure foundation. It would be better to complain of the insufficiency of an adopted medicine, than to connect it with powers and capabilities which, in the moment of trial, it will be found not to possess. The precise influence attributed to various medicines being taken too much on trust, prevents those researches which are necessary for furnishing that description of influence.

In the materia medica, as in other departments of medical science, knowledge cannot be stationary; it must be progressive. What is known at this time may, at a future period, under farther combinations and illustrations, be understood so differently, as greatly to extend the intrinsic worth of the acquisition. It is not in the nature of things that medicinal substances should uniformly produce a similar effect in all constitutions, or even in the same individual, at all times. The variations, the discrepancies, the unfavourable circumstances occurring on one occasion and not on another, must necessarily lead to widely different results. Hence the frequent fallacy of reputed remedies; and hence the physical impossibility of strict uniformity in medicinal influence, and the utter absurdity of confiding in specific nostrums.

Beyond the sphere of the first passages medicinal influence is precarious, and cannot be reasonably regarded as affording any infallible ground on which to estimate its ulterior actions. The sympathies naturally subsisting between the alimentary canal and the system at large, are powerful enough to connect them together, by ties of medicinal as well as nutritive influence. Whether the various impressions produced by different medicinal substances on the stomach and intestines, are transmitted to portions of the system possessing a corresponding susceptibility to be so affected; or whether the substance exerts a direct material agency, by finding admission into the system; is a question involved in too much perplexity for the present state of physiological knowledge satisfactorily to solve. But this uncertainty offers no objection to the fact, that certain parts, whether of visceral, tubular, fibrous, or reticulated arrangements, are as it were specifically affected by the peculiar influence of given agents. The diversified actions of the various substances of the materia medica depend on this principle; and it is in the precise adaptation of the agent to the peculiar susceptibility for being affected by it, that consists the greater or less efficacy of all remedies in accomplishing their curative purposes.

It is most clear, that no medicine can infallibly produce a given effect in the variable state of the animal economy. Whatever harmony or aptitude for a certain influence may exist at one period, will be found disconcerted and inoperative at another; so that nothing but approximations to the precise effect contemplated can generally be expected. If the morbid condition requiring to be remedied be founded in either excessive or deficient excitement, in an habitual or a distempered state of action, in too much or too little susceptibility for being affected by medicinal influence, the appropriate means for correcting either or all of these different morbid states will be more or less efficient, according to inherent circumstances of organization, or to accidental conditions of prevailing excitability. It is only by reiterated trials, close observation, and accumulated results, that a just conclusion can be formed of the real power of medicinal substances, and to what extent their efficacy and fallacy in relieving and curing disease may be safely estimated and allowed.

Digitalis, uva ursi, and acetate of lead, have of late been more particularly regarded as possessing remedial powers in all stages of phthisis pulmonalis. That each and all of them, with many other articles that might be enumerated, are capable of exerting considerable influence in allaying, and perhaps, in certain circumstances, even in subduing, the diseased action existing in that ailment, will not be denied; but that either of them is entitled to the reputation of being specifically efficacious in curing that malady, is a position that may be reasonably doubted. Experience has not hitherto justified the early character respectively assigned to these remedies. They have, no doubt, when assiduously and discriminatively given, severally afforded at least palliative, if not curative, benefit in the recoverable states of pulmonary consumption. But, to suppose that the salutary influence occasionally exerted by them should uniformly take place, would be to imagine a condition of vital similarity in all relative circumstances that but rarely occurs. The deviations from a given standard are so constant, and often so wide, as to preclude all reasonable expectation that the same effect should necessarily arise. Approaches towards it may, and indeed frequently are made, and in the incompleteness of the resemblance is found the want of specific efficacy. The failures are as explicable as the successes, and equally rest on the principle of dissimilarity, more or less influential on medicinal agency obtaining in the excitable conditions of vital power. It is therefore wrong to reject a medicine because it is not uniformly available for the curative objects in view. If it be so in certain instances, it deserves to be retained; and trials should be patiently instituted, to ascertain when, and in what probable manner, its most salutary effects appear to be produced. When a curative indication in the treatment of disease cannot be fulfilled by a medicine usually employed for that purpose, it is possible the deficiency may be supplied by an agent possessing analogous qualities; so that what was not attainable by one mode of power, may be effected by another kindred one, or by a conjunction of their similar virtues. Thus, digitalis will often be more efficacious when united to acetate of lead, and these two when in triple connexion with uva ursi, than when given separately. The practice of conjoining substances in medicinal composition is vindicable on the ground of the insufficiency of single agents to effect the desired object. The power that may be wanting cannot always be supplied by an augmented dose of the same article, but by the combined influence of various substances, each sustaining and extending the other's efficacy.

It is precipitate and unphilosophical to propose any remedy as capable of acting with undeviating sameness of power. The requisites for such an agency have no existence in the animal economy; it is therefore a chimera which adequate trials refute, and never establish. The highest character that can be justly given to a new medicine, is, that it will prove less fallacious than its predecessor in reputation; that it will not so often disappoint expectation; but it must be at the same time allowed, that it can act by no means uniformly in the dissimilar and variable circumstances in which it will have to operate in different constitutions. A medicine should not be abandoned because it cannot effect impossibilities; but should be retained and upheld as an useful acquisition, when it is capable of fulfilling the curative intention with which it is given oftener than any other known medicine could have done. In this estimate of comparative worth, digitalis, cerussa acetata, and uva ursi, deserve to be regarded as valuable medicines; but not of a description to be considered as acting with either precisely similar or infallible efficacy.

The reputation to which the muriates of lime and barytes have severally aspired, for effectually relieving and curing scrofulous disease, has not been warranted by experience. The object to be effected by their influence is too difficult to admit of being realized in a way that would entitle either these or any other agents to much reliance. To correct and repair morbid derangements of the glandular system, and to reinstate that portion of the general frame in a healthy condition, may be anxiously desired, but cannot be readily accomplished. Carbonate of iron, cinchona, soda, cicuta, and arsenic, may respectively lay fair claim to considerable efficacy in scrofulous diseases; but, if any pretension be made to a directly specific or a uniformly curative power, the endeavour to obtain it would prove fallacious. Much reasonable expectation might be indulged as to the salutary influence of these different substances; but the various and uncertain conditions in which they must have to operate, when subjected to trial, must necessarily render the results always too doubtful to admit of unqualified confidence.

Eau medicinale, colchicum, white hellebore, and alkalies, have severally obtained a reputation more or less ephemeral, for relieving and curing gouty irritation. That each of these remedies may so affect the excitability of the system, as in some measure to counteract the painful sensation of arthritic disease, cannot be reasonably disputed; but, that either can be regarded as possessing specific powers in overcoming that description of ailment, is inadmissible, on the same principle that would deny a similar claim to any sort of medicine that could be proposed. It is the extreme uncertainty of this effect that should refer the pretensions of all new remedies to the test of actual observation, guarded by a due consideration of the improbability of the same physical circumstances occurring in the different individuals who may be subjected to their influence. Unvarying uniformity is not known in any of the departments of nature; general resemblances mark the characters of kindred substances; but this analogy is too loose for intrinsic and unexceptionable identity. It is in this inherent dissimilarity in the animal economy amidst an apparent sameness, inducing a persuasion that no influential difference could exist, that consists the ground on which medicinal remedies produce their dissimilar effects under apparently similar circumstances. In this innate organic diversity will be found an adequate explanation of the uncertainty, the insufficiency, and of what would occasionally appear to be the capricious or arbitrary, effects of medicines. To know the susceptibilities of vital action in all their original and acquired power, would be to understand the almost infinite variety of effect that may result from the same medicinal agents. The discordancy which thus presents itself to the observant practitioner, would suggest to him the importance of scrupulous attention to the power of medicine in different persons, that he might, at length, be enabled to come to some general conclusion that would be steadily applicable to the medical purposes of the healing art.

It is hardly conceivable, that direct specific and permanent efficacy will ever be produced by any particular agent, however high its just reputation may be, and with whatever encomiums its introduction to public notice may be accompanied. The deficiencies of one agent should be supplied by the analogous quality of another ; and, in this mode, sufficient remedial power may be gathered from sources that yield similar efficacy. Like all other knowledge, that of medicine is progressive ; and, were that which is worth retaining preferred to the attraction of novelty, and were more assiduity employed in simplifying and classing medicinal influence, it would be soon seen that useful practical acquirements are of slow growth, and can only result from rigid analytical examination and unsparing critical enquiry. Severe and patient observation is necessary, to be enabled correctly to appreciate the value of medicinal efficacy. The existing properties, whether chemical, mechanical, or medicinal, should be investigated and understood, so that a reasonable inference may be drawn of what would be the probable effect of such an agent. Close attention will also detect the constitutional circumstances that may appear to be most favourable to a given medicinal effect. When these relative peculiarities are sufficiently known, much reasonable confidence may be indulged in the usual and probable result. Although unbounded reliance on medicinal agency may savour of empiric confidence, yet a certain extent of expectation may be justly held, without fear of disappointment. When remedies, in a very large majority of instances, on a scale of adequate trial, shall have vindicated their claim to specific efficacy, an authority sufficiently competent is afforded for depending on a continuance of such power. It is by this tenure that all medicines of reputed efficacy are possessed and used. The most valuable remedies known in medical practice have no higher title to credit than the customary manner in which they operate beneficially. Every new medicinal substance should be brought to the test of experiment. It should be cautiously ascertained what it can effect, and in what pretension it is likely to fail. When practical proof has been obtained on its real *bona fide* capability, then the visionary notions of specific and infallible powers in certain states of disease should be corrected and restrained by the consideration, that the sameness of condition which is necessary to insure an unvarying effect does not present; and that, therefore, such inevitable uniformity has no existence in any sort of medicinal operation.

Writers of the highest authority have been unjustly regarded as case-makers, in some of the details which they have given of medicinal efficacy. The results of trials, supposed to be fully sufficient to ascertain all that was necessary to be known, have been published of the remedial powers of various medicines. It is indeed to be regretted, that these accounts have not been verified, in the observation of others, to the extent proposed by the authors of them; yet it would be illiberal to suppose, that they have not been communicated, in most instances, under a clear persuasion of their truth and validity. The incorrectness, if any, on these occasions, would appear to be a too-exclusive reference to the cases in which success had occurred; so that the view given, instead of being a general, was a selected, statement of the favourable issues of the trials that had been instituted. This is a partiality into which an anxious desire to advance the practical improvement of medicine is apt to betray an unguarded enquiry after facts. The statement indeed contains facts; but they are too insulated, and form too desultory a connexion with the whole that should be brought under consideration, to admit of their being at all conclusive of the general efficacy intended to be deduced from them. On these occasions, it would be better to state the facts, and to cite unreservedly the instances of failure as well as of success. All the trials should be mentioned, and all the attending circumstances should be minutely detailed, which would collectively present a correct practical view, not only of what had actually occurred, but of what would be likely invariably to happen in all subsequent enquiries.

The accounts which have been lately published of the curative efficacy of belladonna, in that afflictingly-painful complaint the tic douleureux; of hydrocyanic or prussic acid, in pulmonary affections; and of colchicum, in gouty disease; cannot be wholly destitute of truth. They have, no doubt, respectively operated in a salutary manner; but yet neither of them can be implicitly relied on in the several ailments in which they are held to be curative. All medicinal efficacy should be measured and weighed most scrupulously; the *pro et contra* should be impartially estimated; and the real worth of the evidence be decided by all that is known concerning it. Impatient examinations and hasty conclusions are almost necessarily erroneous; whilst patient enquiry, and cautious induction from indisputable facts, will as inevitably conduce to ascertain and establish truth.

*Taunton; May 7th,* 1820.

